# Expression of serotonin 1A and 2A receptors in molecular- and projection-defined neurons of the mouse insular cortex

**DOI:** 10.1186/s13041-020-00605-5

**Published:** 2020-06-29

**Authors:** Anes Ju, Beatriz Fernandez-Arroyo, Yifan Wu, Débora Jacky, Anna Beyeler

**Affiliations:** Neurocentre Magendie, INSERM 1215, Université de Bordeaux, 146 Rue Léo Saignat, 33000 Bordeaux, France

**Keywords:** Neural circuit, Anxiety, Immunohistochemistry, Retrograde tracing, Neuroanatomy, Excitatory neurons, Inhibitory neurons, Projection neurons, Synaptophysin

## Abstract

The serotonin (5-HT) system is the target of multiple anxiolytics, including Buspirone, which is a partial agonist of the serotonin 1A receptor (5-HT1A). Similarly, ligands of the serotonin 2A receptor (5-HT2A) were shown to alter anxiety level. The 5-HT1A and 2A receptors are widely expressed across the brain, but the target region(s) underlying the influence of those receptors on anxiety remain unknown. Interestingly, recent studies in human and non-human primates have shown that the 5-HT1A and 5-HT2A binding potentials within the insular cortex (insula) are correlated to anxiety. As an initial step to define the function of 5-HT transmission in the insula, we quantified the proportion of specific neuronal populations of the insula expressing 5-HT1A or 5-HT2A. We analyzed seven neural populations, including three defined by a molecular marker (putative glutamate, GABA or parvalbumin), and four defined by their projections to different downstream targets. First, we found that more than 70% of putative glutamatergic neurons, and only 30% of GABAergic neurons express the 5-HT1A. Second, within insular projection neurons, 5-HT1A is highly expressed (75–80%) in the populations targeting one sub-nuclei of the amygdala (central or basolateral), or targeting the rostral or caudal sections of the lateral hypothalamus (LH). Similarly, 70% of putative glutamatergic neurons and only 30% of insular GABAergic neurons contain 5-HT2A. Finally, the 5-HT2A is present in a majority of insula-amygdala and insula-LH projection neurons (73–82%). These observations suggest that most glutamatergic neurons can respond to 5-HT through 5-HT1A or 5-HT2A in the insula, and that 5-HT directly affects a limited number of GABAergic neurons. This study defines a molecular and neuroanatomical map of the 5-HT system within the insular cortex, providing ground knowledge to identify the potential role of serotonergic modulation of selective insular populations in anxiety.

## Introduction

### The serotonin system in anxiety

**Fig. 1:**
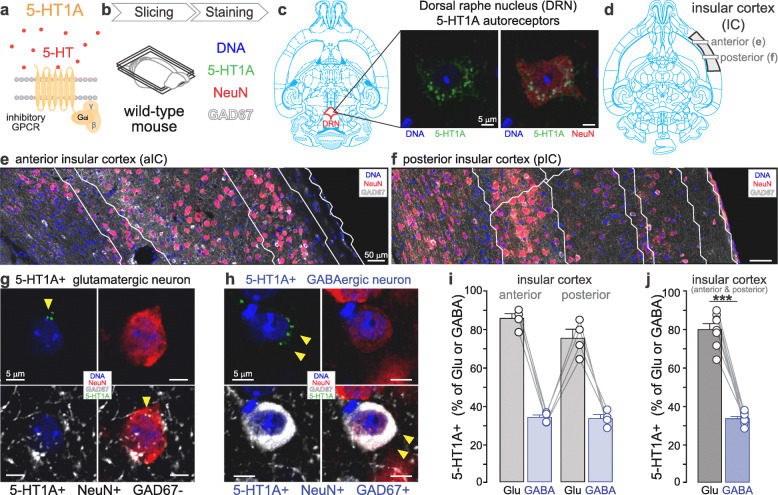
Expression of 5-HT1A in the glutamatergic and GABAergic neurons of the insular cortex. **a.** Molecular diagram of 5-HT1A coupled to an inhibitory G protein. **b.** Experimental design to identify 5-HT1A-expressing (5-HT1A+) glutamatergic and GABAergic neurons in the insula using immunofluorescent staining in horizontal brain slice of wild-type mouse. **c.** 5-HT1A antibody staining of soma 5-HT1A-autoreceptors in neurons of the dorsal raphe nucleus (DRN). **d.** Definition of the analyzed area in the anterior and posterior insula in horizontal brain slices (3.60 mm ventral to Bregma). **e-f.** Image of DNA staining and immunofluorescent signal of NeuN and GAD67 in the anterior (**e**) and posterior (**f**) insula. White lines indicate the division of cortical layers in the insula. **g-h.** Representative images of 5-HT1A+ glutamatergic (**g**) and GABAergic neurons (**h**) in the insula. Yellow arrows indicate 5-HT1A expression. **i.** Percentage of 5-HT1A expression in glutamatergic (Glu) and GABAergic (GABA) neurons in the anterior (ICa) and posterior insula (ICp). Total glutamatergic neurons: ICa + ICp = 1966 + 2359 = 4325; Total GABAergic neurons: ICa + ICp = 513 + 373 = 886; *n* = 4 mice **j.** The proportion of 5-HT1A+ neurons is significantly higher in the glutamatergic than in the GABAergic population (paired t-test, ***p < 0.001), n = 4 mice, 2 locations per mouse

Serotonin (5-hydroxytryptamin; 5-HT) is a neuromodulator that plays a critical role in a wide range of functions including mood, sleep, attention and learning, as well as psychiatric conditions such as anxiety. The involvement of serotonin in the pathophysiology of anxiety disorders has gained interest as selective serotonin reuptake inhibitors (SSRIs) are now used as the first-line treatment for anxiety disorders [1–5]. Within the 5-HT system, along with 5-HT transporters, different 5-HT receptor subtypes, such as the serotonin 1A receptors (5-HT1A), are also targeted by anxiolytics [6]. Specifically, the partial agonist of the 5-HT1A, Buspirone, was the first pharmacotherapeutic alternative to benzodiazepines for the treatment of generalized anxiety disorder [7, 8]. Conversely, in clinical practice, the blockade of serotonin 2A receptor (5-HT2A) has been reported to augment the therapeutic efficacy of SSRIs [9, 10]. Moreover, alterations of anxiety-like behaviors were observed in 5-HT1A or 5-HT2A knock-out mouse lines (*Htr1a* or *Htr2a*) [11, 12]. Collectively, these clinical and preclinical observations suggest that 5-HT1A and 2A receptors are central players in controlling anxiety. The 5-HT1A and 5-HT2A are inhibitory and excitatory G-protein coupled receptors (GPCRs, Fig. 1a and 5a), respectively mediating hyperpolarization and depolarization of neurons expressing those receptors [13–16]. Brain-wide analysis of the localization of these two receptors identified expression across dozens of brain regions (Supplementary Fig. 1, Video 1 and 2), however, the brain structure(s) underlying the anxiolytic effects of these receptors remain to be identified.

### The insular cortex in anxiety

Recently, an increasing number of publications on the human insular cortex has identified this region as a core brain region involved in multiple psychiatric disorders [17]. A meta-analysis of functional magnetic resonance imaging (fMRI) studies in patients with anxiety disorders identified hyperactivity of the insular cortex (insula or IC) compared to healthy controls [18], suggesting that the insula is involved in anxiety. The insula is a wide cortical region, composed of three different areas: the granular, dysgranular and agranular subdivisions, defined by their cytoarchitecture (presence or absence of layer IV) [19]. Interestingly, the insula is also functionally divided into an anterior and a posterior section (ICa and ICp), as demonstrated by recent studies showing that their optogenetic activation produced behavioral responses of positive and negative valence respectively [20]. Regarding anxiety-related behaviors, pharmacological activation of those two insular subregions in rats, respectively induced anxiogenic and anxiolytic effects in the elevated plus maze [21]. However, another recent optogenetic study has reported that close-loop inhibition of glutamatergic neurons of the posterior insula increased exploration of the open arms in the elevated plus maze [22]. Collectively, data from patients and animal models have identified the insular cortex as an important brain region involved in anxiety [17].

### Serotonin 1A and 2A receptors in the insular cortex

Immunostaining of the serotonin transporter (SERT) has shown that the insula contains dense serotonergic afferents [23] and brain-wide in situ mRNA hybridization has identified expression of 5-HT1A and 2A receptors in the insula (Supplementary Fig. 1 and Video 1–2, http://mouse.brain-map.org/) [24]. Very interestingly, imaging studies using positron emission tomography (PET) have demonstrated a reduction of the binding potential of 5-HT1A in the insula of patients diagnosed with social anxiety disorder [25] as well as in the insula of stressed individuals [26]. Similarly, another PET study has identified a negative correlation between 5-HT2A binding potential and trait anxiety in non-human primates (marmosets) [27]. These data suggest that the insular cortex is, at least partially, underlying the anxiolytic effect of the serotonergic pharmacotherapies used to treat anxiety disorders.

### Insular cortex connectivity

Seminal anatomical studies in rodents have revealed that insular projection neurons target a variety of subcortical structures, including the amygdala and the lateral hypothalamus (LH) [22, 28, 29]. Further studies in animal models showed that projections of the anterior insula to the basolateral amygdala (ICa-BLA) and posterior insula to central amygdala (ICp-CeA) are involved in positive and negative valence respectively [29]. Importantly, studies in humans assessing the functional connectivity between the insula and the amygdala have shown that their connectivity is increased in patients with anxiety disorders [30, 31]. A recent study showed that optogenetic stimulation of ventral hippocampus terminals in the LH increases anxiety, suggesting that along the amygdala [32], the LH is part of the brain circuits modulating anxiety [33].

### Research objective

While both 5-HT1A and 5-HT2A are reported to be expressed in glutamatergic and GABAergic neurons of the mouse prefrontal cortex [34], the proportion of those neuronal populations expressing 5-HT1A or 2A receptors in the insular cortex remains unknown. Moreover, the proportion of neurons expressing 5-HT1A or 2A receptors within neural populations defined by their downstream targets has not been investigated. Based on the involvement of 5-HT1A and 2A receptors in anxiety, along with the implication of selective insular pathways, we hypothesized that those two receptors are differentially expressed in specific neural populations in the insula. We analyzed seven neural populations defined by a molecular marker (putative glutamate, GABA or parvalbumin), or defined by their projection to one of four different downstream targets. Using immunohistochemistry we quantified the proportion of insula pyramidal and GABAergic neurons expressing 5-HT1A or 2A receptors in mice, in both anterior and posterior insula. Using a transgenic mouse line, and by combining immunochemistry with retrograde tracing, we then quantified the proportion of projection neurons expressing 5-HT1A or 2A in insular populations targeting different territories of the amygdala and the lateral hypothalamus.

## Methods

### Animals

Adult male and female mice of 10 weeks old were used for experiments. Eleven male and eight female wild-type C57BL/6 J (Charles River Laboratory), four male Htr1a-IRES2-Cre-D knock-in mice (*Htr1a-Cre*; 030160, Jax Laboratory) as well as four male PVa-cre::Ai14 (PVa-cre; 008069, Jax Laboratory, Ai14; 007914, Jax Laboratory) were used in this study. Animals were kept group-housed (4–6 animals per cage) in a controlled environment (reverse 12 h light/dark cycle) with ad libitum food and water. All procedures of handling animals were approved by the French government (Ministère de lʼEnseignement Supérieur, de la Recherche et de lʼInnovation, Saisine #20414 and #19355,) and in accordance with the guidelines of the European Communities Council Directives.

### Stereotaxic surgery

Mice were anesthetized with isoflurane (5% for induction, 1.5–2.0% afterward) in the motorized stereotaxic frame (MTM-3, WPI) for the entire surgery and their body temperature was maintained with a heating pad. In order to label the insular neurons projecting to the downstream targets of our interest, 0.4% of retrograde fluorescent tracer, Cholera toxin subunit B (CTB) coupled to Alexa Fluor 555 or 647 (CTB-555 or CTB-647, Thermo Fisher Scientific, C34776 or C34778), were injected into the basolateral amygdala (BLA, 200 nL), central nuclei of amygdala (CeA, 100 nL), rostral LH (rLH, 200 nL) and caudal LH (cLH, 200 nL) of the right hemisphere of the mouse brain (Fig. 3b and Fig. 4b). The following anteroposterior (AP) / mediolateral (ML) / dorsoventral (DV) stereotaxic coordinates were used (mm from Bregma): BLA: − 1.45 ± 0.15 / + 3.30 / -4.90, CeA: − 0.80 / + 2.35 / -5.20, rLH: − 0.70 / + 1.00 / -5.25, cLH: − 1.70 / + 1.00 / -5.25.

To identify the synaptic targets of 5-HT1A-expressing (5-HT1A+) insula neurons, we injected 200 nL of a mixture of two adeno-associated viral vectors (AAVs, 1:1) into the anterior or posterior insula of *Htr1a-Cre* mice. The first construct was cre-dependently expressing mCherry (AAV_9_-Syn1-DIO-mCherry-WPRE, Vector Builder AAV_9_MP(VB181107-1041gwu)) while the second cre-dependently expressed synaptophysin fused to the enhanced yellow fluorescent protein eYFP (AAV_9_-EF1a-DIO-Syp-EYFP-WPRE, Vector Builder AAV_9_MP(VB181107-1041gwu)). In order to confirm this synaptophysin signal, we used an inverse viral strategy to express synaptophysin-mCherry in 5-HT1A-expressing insula neurons. We injected 250 nL of a mixture of two AAVs (1:2) into the anterior or posterior part of the insula of Htr1a-Cre mice. One construct cre-dependently expressed synaptophysin-mCherry (AAV8/2-hEF1a-DIO-Syp-mCherry, MGH, AAV-RN1) and the other construct cre-dependently expressed eYFP (AAV9-EF1a-DIO-eYFP-WPRE-hGH, UPENN, AV-9-27,056). The following AP / ML / DV coordinates were used (mm from Bregma): anterior insula: + 1.70 / + 3.10 / -3.50, posterior insula: − 0.35 / + 4.20 / -4.20.

Injections were performed using glass pipettes (3–000-203-G/X, Drummond) made by a puller (PC-100, Narishige) to deliver the retrograde tracer or viral vector at a rate of 5–8 nL/s using a Nanoject III (3–000-207, Drummond). After completion of the injection, the pipette was raised 100 μm, left for additional 10 min to allow diffusion of the retrograde tracer or the virus at the injection site, and then slowly withdrawn. After surgery, the mouse body temperature was maintained using a heat lamp until the animal fully recovered from anesthesia. Mice returned to their home cages for 1 week to allow CTB retrograde transport or 4 weeks to allow viral expression.

### Immunohistochemistry

Brains were perfused and post-fixed with 4% paraformaldehyde (antigenfix, F/P0014, MM France) at 4 °C overnight, and cryo-protected in 30% sucrose in PBS. After embedded in optimum cutting temperature compound (OCT), horizontal sections (50 μm thick) or coronal sections (50 μm thick) were obtained using a frozen sliding microtome (12062999, Thermo Scientific) and stored in PBS at 4 °C until processing for histology.

As these brain slices contain both anterior and posterior insula, the horizontal sections 3.60 mm ventral to Bregma were used for immunostaining (Fig. 1d). Two successive brain sections with 50 μm dorsoventral difference were chosen and one section per brain was used for 5-HT1A staining and counting, and the next section was used for 5-HT2A staining and counting. After washed in PBS, the brain sections were blocked with 10% normal goat serum (NGS) and 0.5% Triton X-100 in PBS for 1 h at room temperature. 5% NGS and 0.5% Triton X-100 in PBS was also used for the primary and secondary antibody dilution. Guinea pig polyclonal anti-NeuN (1:500; 266004, SYSY) [35], mouse monoclonal anti-GAD67 (1:500; MAB5406, Millipore) [36–38], rabbit polyclonal anti-5-HT1A receptor (1:500; ADI-905-741, Enzo) [39] and rabbit polyclonal anti-5-HT2A receptor (1:250; ab66049, Abcam) [40–44] were used as primary antibodies. The primary antibodies used in this study were validated and widely used in previous publications [35–44]. Alexa Fluor 488 goat anti-rabbit IgG (A11034, Fisher Scientific), Alexa Fluor 555 goat anti-guinea pig IgG (A21435, Fisher Scientific) and Alexa Fluor 647 goat anti-mouse IgG (A21236, Fisher Scientific) were used as secondary antibodies. Incubation of the primary antibodies was carried out at 4 °C overnight, followed by incubation of secondary antibodies for 3 h at room temperature with 1:1000. Washing using PBS for 5 min × 3 times was performed between every step. Lastly, Hoechst33342 (written DNA in the figures, 1:5000; 11544876, Fischer scientific) diluted in PBS was applied on the slice for 15 min before coverslips were applied.

### Imaging and cell counting

Images were captured through a 10x dry objective (NA 0.70), a 40x oil-immersed objective (NA 1.30) and a 63x oil-immersed objective (NA 1.40) from a Leica SP8 confocal microscope (Leica Microscopy). First, all processed brain slices, as well as all slices containing an injection site were imaged using 10x or 20x objectives. For cell counting of NeuN, GAD-67, PV and insula projectors, z-stacks of ROIs (z step: 0.5 μm, 3–4 frame average, 1024 × 1024 pixels) were scanned using the 40x objective (Fig. 1e-f and 3c and Supplementary Fig. 2a). Injection of retrograde tracers in two different targets is not an appropriate technique to quantify collateralization to two downstream regions due to three main limitations: (1) tracers have limited efficiency in retrograde transport (2) different CTB retrograde tracers have different efficiencies and (3) injections are performed to prioritize specificity over coverage of the target regions [45]. However, the overlap of CTB-555 and CTB-647 can instruct us whether collateralization exists. Additionally, we detected few, but some cell bodies containing both CTB-555 and CTB-647. Specifically, we found that 3.84 ± 1.35% of insula-amygdala labeled neurons were both IC-BLA and IC-CeA projectors (*n* = 5 mice, data not shown), and that 3.70 ± 0.14% of insula-LH labeled neurons were both IC-rLH and IC-cLH projectors (*n* = 4 mice, data not shown). Therefore, neurons projecting to both BLA and CeA, or to both rLH and cLH exist, but their exact proportion should be studied using different methods, such as synaptophysin expression using viral vectors [45]. Example immunofluorescent pictures of 5-HT1A+ or 5-HT2A+ cells were captured with the 63x objective (Fig. 1g-h, 3d-e, 5c-d and 5g-h and Supplementary Fig. 2b-c). In order to detect synaptophysin-eYFP or synaptophysin-mCherry signal, z-stacks of BLA, CeA, rLH and cLH have been scanned with 63x objective in a picture format of 1024 × 1024 pixels (Number of z-stacks: 30, z-steps: ~ 1 ± 0.10 μm, 2–3 frame average). All images have been processed using the open source Fiji software (ImageJ, NIH), and cell counting has been performed manually.

### Brain atlases

Three different atlases of the mouse brain were used in this study. Data of mRNA expression level in different brain regions were extracted from the Allen Mouse Brain Atlas (Supplementary Fig. 1, Video 1 and 2, http://mouse.brain-map.org/). The mRNA expression level in regions of interests (ROIs) of in situ hybridization was calculated by multiplying the expression density and intensity [24, 46].

$$ mRNA\ expression\ level= expression\ density\times expression\ intensity $$

$$ Expression\ density=\frac{\sum expressing\ pixel\ in\  ROI\ }{\sum all\  pixels\ in\  ROI} $$

$$ Expression\ intensity=\frac{\sum expressing\ pixel\ in tensity\ in\  ROI}{\sum expressing\ pixel s\ in\  ROI} $$

**Fig. 2:**
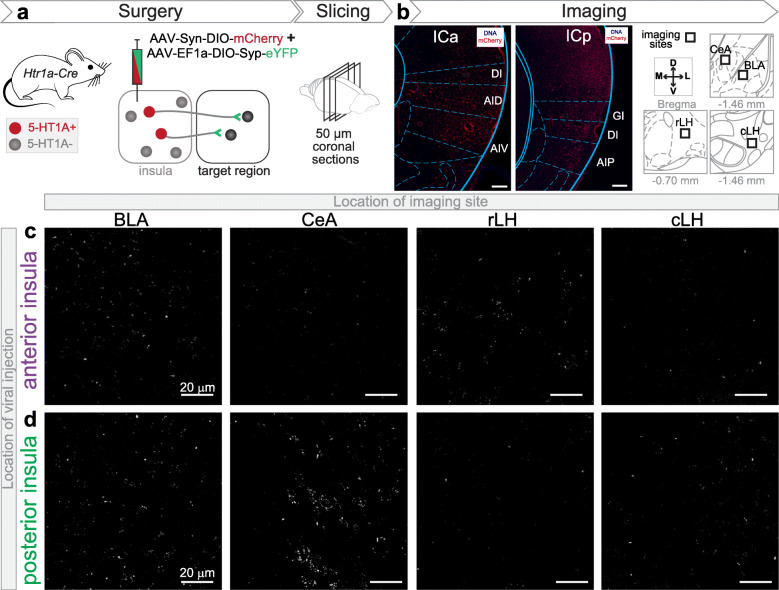
Evidence of synaptic contacts of 5-HT1A+ insular neurons in the amygdala and lateral hypothalamus. **a.** Experimental design to detect synaptic terminals of 5-HT1A-expressing (5-HT1A+) insula neurons through cre-dependent expression of mCherry and synaptophysin-eYFP in the anterior or posterior insula of *Htr1a-Cre* mice. **b.** (**Left**) Confocal image of the cre-dependent viral vector injection site in a coronal section of the anterior (DI: dysgranular insula, AID: agranular insula dorsal part, AIV: agranular insula ventral part) and the posterior insula (GI: granular insula, AIP: agranular insula posterior part). Note mCherry expression in the soma of 5-HT1A+ neurons. (**Right**) Imaging locations of synaptophysin-eYFP in the basolateral and central amygdala (BLA and CeA) as well as the rostral and caudal part of the lateral hypothalamus (rLH and cLH). Distances are in the anteroposterior axis from Bregma. **c-d.** Confocal images of the expression of synaptophysin-eYFP in BLA, CeA, rLH and cLH, originating from 5-HT1A+ neurons of the anterior (**c**) and posterior (**d**) insula

**Fig. 3:**
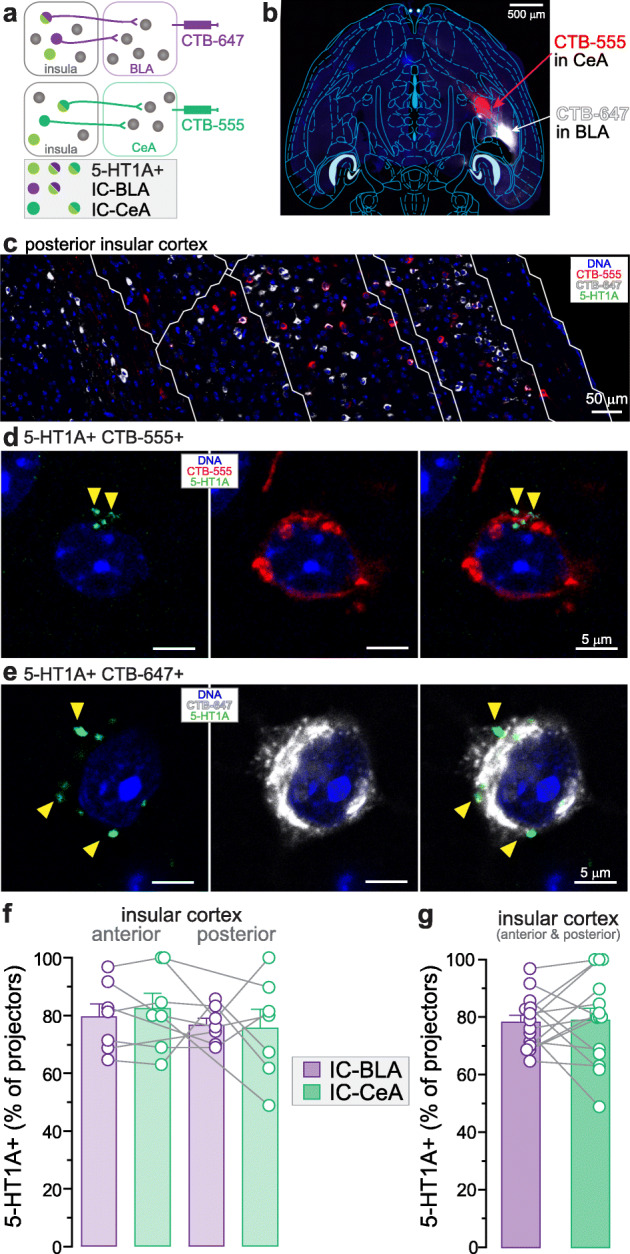
Expression of 5-HT1A in insula neurons projecting to subnuclei of the amygdala. **a.** Experimental timeline combining retrograde fluorescent tracers (CTB) injections and 5-HT1A immunostaining. **b.** Injection site of retrograde fluorescent tracers (CTB-555 and CTB-647) in the central and basolateral amygdala (CeA and BLA) within a horizontal brain slice (4.56 mm ventral to Bregma). **c.** Immunofluorescent image showing CTB-555 and CTB-647 positive neurons in the posterior insula. **d-e.** Representative fluorescent images of CTB-labelled neurons expressing 5-HT1A in the insula. Yellow arrows indicate 5-HT1A expression. **f.** Percentage of 5-HT1A expression in neurons projecting to the BLA and CeA in the anterior (ICa) and posterior insula (ICp). **g.** The proportion of 5-HT1A+ neurons is not different between IC-BLA and IC-CeA populations (unpaired t-test, *p* > 0.05). Total IC-BLA neurons: ICa + ICp = 417 + 620 = 1037; Total IC-CeA neurons: ICa + ICp = 455 + 647 = 1102; *n* = 7 mice, 2 locations per mouse

**Fig. 4:**
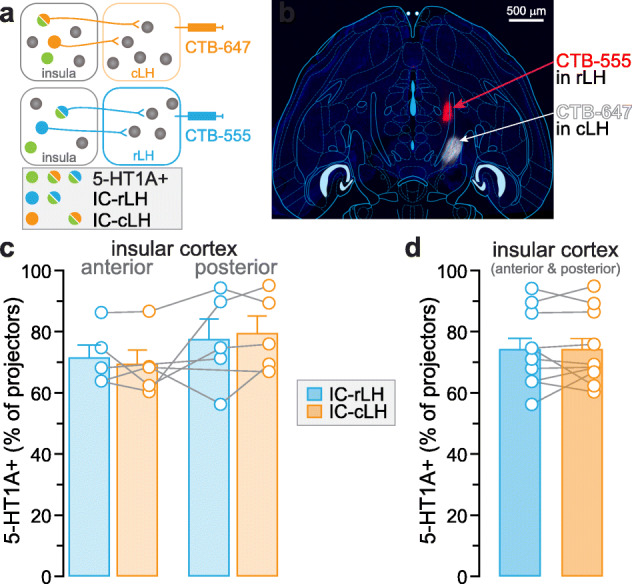
Expression of 5-HT1A in insular neurons projecting to the lateral hypothalamus. **a**. Experimental design combining retrograde fluorescent tracers (CTB) and serotonin 1A receptor (5-HT1A) antibody staining. **b.** Injection site of retrograde fluorescent tracers (CTB-555 and CTB-647) in the rostral and caudal parts of lateral hypothalamus (rLH and cLH, respectively) within a horizontal brain slice (4.56 mm ventral to Bregma). **c.** Percentage of 5-HT1A expression in neurons projecting to cLH and rLH from the anterior (ICa) and posterior (ICp) insula. **d**. The proportion of 5-HT1A+ neurons are not different between IC-rLH and IC-cLH populations (unpaired t-test, p > 0.05). Total IC-rLH neurons: ICa + ICp = 298 + 313 = 611; Total IC-CeA neurons: ICa + ICp = 422 + 244 = 666; *n* = 5 mice, 2 locations per mouse

The Franklin & Paxino’s Brain Atlas was used to confirm the injection sites of CTB- or virus-injected brains (Fig. 2a, 3b, and 4b) and to determine the scanning regions in the insular cortex (Fig. 1d) and the dorsal raphe nucleus (Fig. 1c) [47].

To compute the overall proportions of 5-HT1A and 2A expressing neurons among insular neurons (Fig. 6b), we calculated the percentage of glutamatergic and GABAergic neurons using the Blue Brain Cell Atlas (https://bbp.epfl.ch/nexus/cell-atlas/) [48]. This recent study provides the first 3D cell atlas of the entire mouse brain, providing cell positions algorithmically constructed from Nissl staining and in situ hybridization of specific genetic markers within the whole brain. We included the total number of neurons, of excitatory neurons and of inhibitory neurons in the entire insular cortex, including: gustatory areas layer 1, 2/3, 4 5 6a and 6b, as well as dorsal, posterior and ventral agranular insular area. From their database, 73% of insular neurons were defined as excitatory (glutamatergic) and 27% were defined as inhibitory (GABAergic).

### Statistical analysis

Histogram bars represent the mean ± SEM and were plotted in Prism 7 software (Graphpad). Two-tailed non-parametric t-test was used to compare percentages of glutamatergic and GABAergic neurons expressing 5-HT1A and 2A receptors in the insula. Statistical significance was defined at a *p*-value lower than 0.05. *p < 0.05, **p < 0.01, ***p < 0.001.

## Results

### 5-HT1A is expressed in a higher proportion of insula glutamatergic than insula GABAergic neurons

To analyze the percentage of glutamatergic and GABAergic neurons expressing 5-HT1A in the insula, we performed triple immunofluorescent staining in brain slices of wild-type mice, to label the neuronal marker NeuN, the somatic marker of GABAergic neurons Glutamate-decarboxylase-67 (GAD 67), and the 5-HT1A receptor (Fig. 1b). To confirm the specificity of 5-HT1A antibody, we imaged *the* signal 5-HT1A autoreceptor which is highly expressed in the neuronal soma of the dorsal raphe nucleus (DRN) [49]. As this antibody stained dense expression of 5-HT1A in the DRN (Fig. 1c), we proceeded with immunofluorescent staining using this antibody to detect the expression of 5-HT1A heteroreceptors, which are located in the soma of postsynaptic neurons [50, 51].

In order to identify glutamatergic and GABAergic neurons in the insula, we used NeuN to label all neurons and GAD67 to label the soma of GABAergic neurons [48]. Based on the fact that the cortical areas are composed glutamatergic and GABAergic neurons [48, 52], putative glutamatergic neurons were detected as NeuN+ and GAD67(−) neurons, whereas GAD67+ neurons were classified as GABAergic neurons (Fig. 1g-h). We found similar percentages of glutamatergic and GABAergic neurons expressing 5-HT1A in the anterior insula (Glu: 85.65 ± 2.5%, GABA: 33.8 ± 1.4%) and posterior insula (Glu: 75.4 ± 4.6%, GABA: 33.3 ± 2.0%, Fig. 1i). Additionally, there was no significant difference in the percentage of 5-HT1A+ glutamatergic and GABAergic neurons between the insular cortex of male and female (Supplementary Fig. 3a). By averaging the data of the anterior and posterior insula of the four mice, we found that 80.5 ± 3.1% of putative glutamatergic and 33.5 ± 1.1% of GABAergic neurons express 5-HT1A (Fig. 1j), indicating 5-HT1A expression in a majority of putative glutamatergic neurons and in a smaller proportion of GABAergic neurons.

Interestingly, serotonin modulates prefrontal synchrony through fast-spiking GABAergic neurons which are known to express 5-HT1A or 2A [53]. To identify whether insula parvalbumin-expressing neurons (PV+) which are fast-spiking GABAergic neurons [54] also express those receptors, we analyzed the percentage of PV+ neurons expressing 5-HT1A+ using immunofluorescent staining of 5-HT1A in the insula of PV-cre::Ai14 mice (Supplementary Fig. 2a-b). 5-HT1A was detected in similar percentages of PV+ neurons in the anterior (29.2 ± 1.7%) and posterior insula (38.3 ± 1.7%, Supplementary Fig. 2d) and overall, 33.8 ± 2.0% of PV+ neurons in the insula expressed 5-HT1A (Supplementary Fig. 2e).

### 5-HT1A is expressed in insula-amygdala and insula-lateral hypothalamus circuits

We hypothesized that 5-HT1A+ neurons in the insula are targeting specific downstream brain regions. According to previous studies, insular neurons are projecting to the basolateral (BLA) and central amygdala (CeA), as well as to the rostral and lateral parts of lateral hypothalamus [22, 29, 55]. As these brain regions are involved in anxiety, we analyzed the synaptic terminals of 5-HT1A+ insular neurons in those regions to identify whether 5-HT1A+ insular neurons make synaptic contacts within these four downstream targets. To label long-range synaptic targets of 5-HT1A+ neurons of the insula, we used *Htr1a-Cre* mice which were designed to express Cre recombinase under the promoter/enhancer sequences of the *Htr1a* gene which codes for the 5-HT1A receptor. We injected a mix of two cre-dependent adeno-associated viral vectors (AAV), one expressing the fluorescent protein mCherry to label the soma of transfected neurons for confirmation of injection sites, and a second one expressing synaptophysin fused to the fluorescent protein (eYFP) in the anterior or posterior insula of *Htr1a-Cre* mice (Fig. 2a) [56]. Additionally, in order to ensure the accuracy of synaptophysin signals, we ran another set of experiments by injecting a mixture of two cre-dependent AAVs: one expressing eYFP and a second one expressing synaptophysin fused to mCherry in the anterior or posterior parts of the insula of *Htr1a-Cre* mice (Supplementary Fig. 5a) Due to its expression in presynaptic terminals, the synaptophysin-eYFP or synaptophysin-mCherry signal can be used as a marker for synaptic contacts [45, 57]. After confirming the injection sites in the anterior or posterior insula (Fig. 2b and Supplementary Fig. 5b), we detected synaptophysin signal in the amygdala and LH (Fig. 2c-d and Supplementary Fig. 5e-f), indicating 5-HT1A+ neurons of both anterior and posterior insular cortex make synaptic contact into the BLA, CeA, rLH and cLH.

We then examined the percentage of specific population of insula-amygdala and insula-LH projection neurons containing 5-HT1A by combining retrograde tracing together with immunofluorescent staining against 5-HT1A (Fig. 3a and 4a). First, CTB-555 or CTB-647 were injected in the BLA or CeA of wild-type mice (counter-balanced across experiments, Fig. 3a). As observed in glutamatergic neurons of the insula, 5-HT1A signal was detected in most of the anterior or posterior insula projection neurons targeting the BLA or CeA (Fig. 3d-f). Overall, 78.2 ± 2.5% of IC-BLA neurons and 79.0 ± 4.2% of IC-CeA neurons express 5-HT1A (Fig. 3g). Second, we injected the retrograde tracers in the rostral and caudal LH (rLH and cLH) and the percentage of 5-HT1A+ neurons projecting to the rLH or cLH was calculated in the anterior and posterior insula (Fig. 4a-c). 5-HT1A expression was detected in 74.1 ± 3.9% of IC-rLH neurons and 74.1 ± 3.8% of IC-cLH neurons (Fig. 4d). No sex difference was found in the percentage of 5-HT1A+ projection populations in the insula (Supplementary Fig. 3b).

### 5-HT2A is expressed differentially in insula glutamatergic and GABAergic neurons, but uniformly in insula-amygdala and insula-LH projectors

**Fig. 5:**
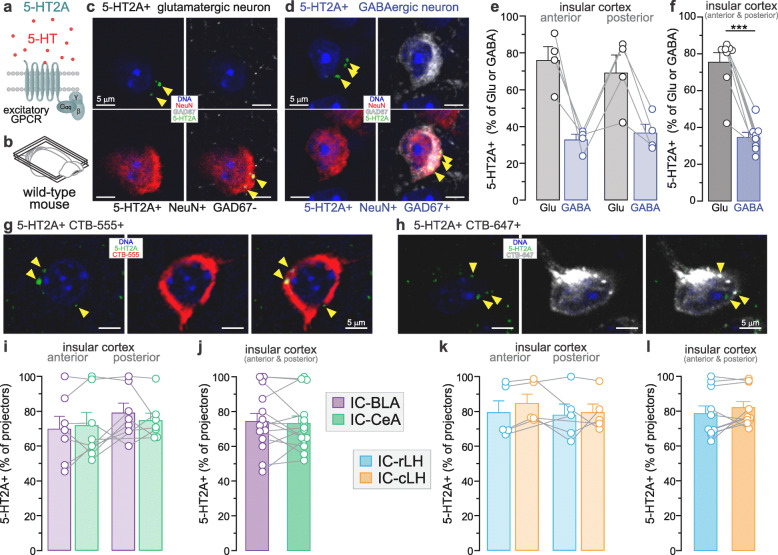
Expression of 5-HT2A in glutamatergic, GABAergic and projection-specific populations of the insular cortex. **a.** Molecular diagram of the serotonin 2A receptor (5-HT2A) coupled to an excitatory G-protein. **b.** Horizontal brain slice of wild-type mouse used in immunofluorescent staining. **c-d.** Representative images of 5-HT2A-expressing (5-HT2A+) glutamatergic (**c**) and GABAergic neurons (**d**) in the insula. Yellow arrows indicate 5-HT2A expression. **e.** Percentage of 5-HT2A expression in the glutamatergic (Glu) and GABAergic (GABA) neurons in the anterior (ICa) and posterior (ICp) insula. **f.** The proportion of 5-HT2A+ neurons is significantly higher in glutamatergic than GABAergic populations (paired t-test, ***p < 0.001). Total glutamatergic neurons: ICa + ICp = 2444 + 2783 = 5227; Total GABAergic neurons: ICa + ICp = 542 + 577 = 1119; *n* = 4 mice. **g-h.** Representative fluorescent images of CTB-labelled neurons (IC-BLA and IC-CeA projection neurons) expressing 5-HT2A in the insula. Yellow arrows indicate 5-HT2A expression. **i.** Percentage of 5-HT2A expression in neurons projecting to the central and basolateral amygdala (CeA and BLA) in the anterior and posterior insula. **j.** The proportions of 5-HT2A+ neurons are not different between IC-BLA and IC-CeA populations (unpaired t-test, p > 0.05). Total IC-BLA neurons: ICa + ICp = 424 + 324 = 748; Total IC-CeA neurons: ICa + ICp = 526 + 523 = 1049; n = 7 mice, 2 locations per mouse. **k.** The percentage of 5-HT2A expression in neurons projecting to rostral and caudal parts of the lateral hypothalamus (rLH and cLH) in the anterior and posterior insula. **l**. The proportions of 5-HT2A+ neurons are not different between IC-rLH and IC-cLH populations (unpaired t-test, p > 0.05). Total IC-rLH neurons: ICa + ICp = 530 + 330 = 860; Total IC-CeA neurons: ICa + ICp = 563 + 259 = 822; n = 5 mice, 2 locations per mouse

The 5-HT2A, coupled to an excitatory G protein (Fig. 5a), is abundantly expressed in cortical regions (Supplementary Fig. 1b, and Supplementary Video 2) and has been suggested to be involved in anxiety. This led us to investigate the percentage of glutamatergic and GABAergic neurons expressing 5-HT2A in the insula. 5-HT2A signal was detected in both glutamatergic and GABAergic neurons (Fig. 5c-d) in similar proportion between the anterior insula (Glu: 76.1 ± 7.3%, GABA: 32.8 ± 3.0%) and posterior insula (Glu: 69.1 ± 9.8%, GABA: 36.5 ± 4.8%, Fig. 5e). As observed for 5-HT1A, 5-HT2A is expressed in a large proportion of the glutamatergic population (72.6 ± 5.8%) and a smaller proportion of the GABAergic population (34.6 ± 2.7%, Fig. 5f). Moreover, PV+ neurons, which are a subtype of GABAergic neurons, contained 5-HT2A in similar proportion in the anterior and posterior insula (Supplementary Fig. 2d), with an average of 31.8 ± 2.3% of PV+ neurons expressing 5-HT2A (Supplementary Fig. 2e). To calculate the percentage of 5-HT2A+ insular neurons targeting the BLA, CeA, rLH and cLH, we performed 5-HT2A antibody staining in horizontal slices of CTB-injected mice (Fig. 3b and 4b). 5-HT2A signal was detected in neurons of the anterior and posterior insula targeting BLA or CeA (Fig. 5g-h). Insular neurons projecting to BLA, CeA, rLH and cLH expressed 5-HT2A in similar proportion in the anterior and posterior insula (Fig. 5i and k). Specifically, 74.4 ± 4.6% of IC-BLA, 73.2 ± 4.2% of IC-CeA, 78.7 ± 4.4% of IC-rLH, and 82.0 ± 3.5% of IC-cLH neurons expressed 5-HT2A (Fig. 5j and l). The neuronal populations including putative glutamatergic, GABAergic and four types of projection neurons expressed 5-HT2A in similar percentage between the male and female insula (Supplementary Fig. 3c-d).

### 5-HT1A and 2A are expressed in similar proportions in the insula

**Fig. 6:**
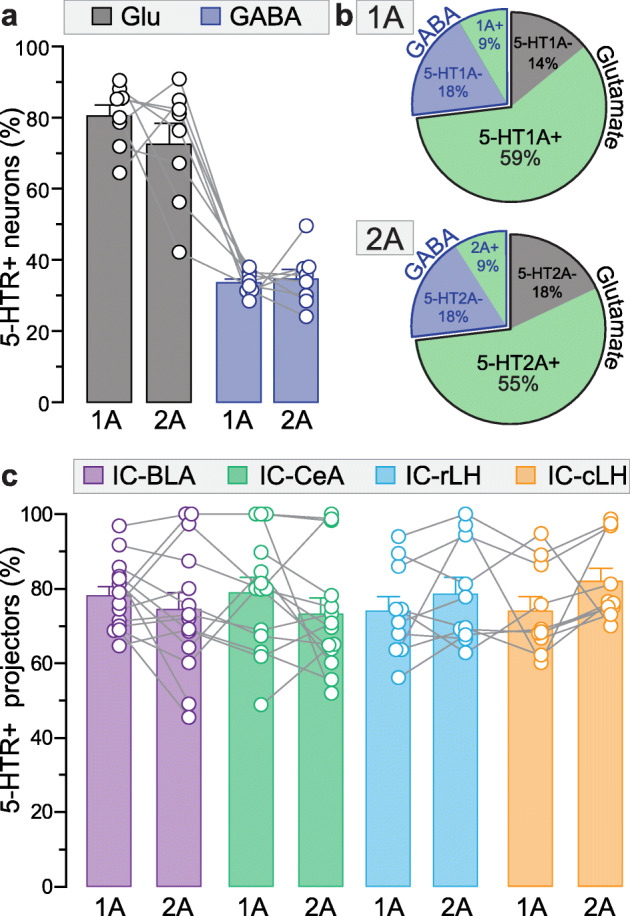
Summary of the proportions of 5-HT1A and 5-HT2A positive neurons in six populations of the insular cortex. **a.** Percentage of glutamatergic (Glu) and GABAergic (GABA) neurons expressing 5-HT1A or 5-HT2A in the insula (data of Fig. 1j and 5f; 1AR: Glu: 4325 cells, GABA: 886 cells; 2AR: Glu: 5227 cells, GABA: 1119 cells, n = 4 mice; 2 locations per mouse). **b.** Percentage of glutamatergic and GABAergic neurons containing, or not containing, 5-HT1A (upper) or 5-HT2A (lower) among total insula neurons. **c.** Percentage of 5-HT1A or 5-HT2A-expressing insular neurons projecting to the basolateral amygdala (IC-BLA), central amygdala (IC-CeA), and rostral or caudal parts of lateral hypothalamus (IC-rLH and IC-cLH, data of Fig. 3g, 4d, 5j and l; 1AR: IC-BLA: 1037 cells, IC-CeA: 1102 cells, n = 7 mice, IC-rLH: 611 cells, IC-cLH: 666 cells, n = 5 mice; 2AR: IC-BLA: 748 cells, IC-CeA: 1049 cells, n = 7 mice, IC-rLH: 860 cells, IC-cLH: 822 cells, n = 5 mice; 2 locations per mouse)

By comparing the percentages of neurons within insular populations which express the 5-HT1A or 2A receptors, we revealed that they are expressed in similar proportions within glutamatergic, GABAergic, IC-BLA, IC-CeA, IC-rLH as well as IC-cLH populations (Fig. 6a and c). Using the estimated numbers of neurons in the mouse insula (total, glutamatergic and GABAergic [48]), we computed the total percentage of neurons expressing and non-expressing 5-HT1A or 2A receptors (Fig. 6b). Overall, more than 50% of insular neurons are glutamatergic 5-HT1A+ neurons (Fig. 6b, top chart). Similarly, half of insular neurons are glutamatergic 5-HT2A+ neurons (Fig. 6b, bottom chart). In comparison, about 9.0% of insular neurons are GABAergic 5-HT1A+ while 9.3% of insula neurons are GABAergic 5-HT2A+ neurons (Fig. 6b). Overall, 68% of insular neurons can be modulated via 5-HT1A, and 64% through 5-HT2A, suggesting an important serotonergic control of the insula function in the rodent brain.

## Discussion

The present study reports the percentage of three molecular-defined and four circuit-defined neuronal populations of the insula, which express 5-HT1A or 5-HT2A. The 5-HT1A is expressed in a large portion of putative glutamatergic neurons (> 70%) and in a small portion of GABAergic neurons (~ 30%), as well as PV+ neurons of the insula. Moreover, a majority (~ 75%) of the insula-amygdala and insula-LH projection neurons contain 5-HT1A. Likewise, 5-HT2A was detected in putative glutamatergic and GABAergic neurons of the insula, as well as in long-range projection neurons targeting the amygdala or LH, with similar proportion as 5-HT1A. Our data support a serotonergic modulation of insula neurons and their circuits through 5-HT1A and 5-HT2A.

### Expression of 5-HT1A and 2A receptors in excitatory neurons

We identified that compared to the GABAergic population, the glutamatergic neural population has a higher proportion of neurons expressing 5-HT1A or 2A receptors in the mouse insula. This observation is consistent with previous in situ hybridization studies within the prefrontal cortex (PFC) of different species, including humans, non-human primates and rodents, showing expression of 5-HT1A or 2A receptors in a high percentage of glutamatergic neurons (~ 60%) but only in ~ 20% of GABAergic cells [34, 58, 59]. Interestingly, in vivo electrophysiological recordings in the PFC suggest that the response of pyramidal neurons to endogenous 5-HT is mainly driven by 5-HT1A and 2A receptors [60]. Given the connectivity of the insula [61], the expression of 5-HT1A and 2A receptors may modulate the serotonergic control of the insular cortex output to other brain regions.

### Expression of 5-HT1A and 2A receptors in inhibitory neurons

Our analyses of the insular cortex show that the expression of 5-HT1A and 5-HT2A are not restricted to putative glutamatergic neurons and are also expressed in GABAergic neurons, although to a smaller extent. Interestingly, in the PFC, despite a similar proportion of GABAergic neurons expressing those receptors, 5-HT1A+ or 5-HT2A+ GABAergic neurons have a pivotal role in finely tuning the activity of neural circuits [53]. Contrary to previous studies showing 5-HT1A-induced hyperpolarization in pyramidal neurons, recent ex vivo electrophysiological recordings in the medial PFC have revealed that bath application of 5-HT1A agonists (8-OH-DPAT) resulted in an increased firing rate of pyramidal neurons, which was blocked by pharmacological application of GABA_A_ receptor antagonist (Gabazine). This result suggests a disinhibitory mechanism, secondary to a reduction of inhibitory interneuron firing [62]. As only 20% of inhibitory cells express 5-HT1A in the PFC, this suggests that the 30% of GABAergic 5-HT1A+ neurons of the insular cortex might also support a disinhibitory effect on the overall activity of the insula.

Interestingly, we found that 30% of PV+ neurons, which are generally fast-spiking neurons, express the 5-HT1A or 2A. Previous studies demonstrated that in the PFC 36% of PV-expressing fast-spiking interneurons contain the 5-HT1A and 23% of PV+ neurons expressed the 5-HT2A, which is consistent with our result [53, 59]. Moreover, in vivo electrophysiological recordings in the PFC of rats under anesthesia combined with electrical stimulation of serotonergic neurons in the dorsal raphe nucleus has identified a serotonergic modulation of slow and gamma oscillations [53]. Specifically, in the PFC of vGAT-Venus rats, single-neuron recordings paired with pharmacological application of 5-HT1A or 2A antagonists, followed by Neurobiotin, PV and 5-HT1A and 2A staining, showed that the frequency and amplitude of slow waves are modulated by 5-HT2A, while fast-spiking interneurons expressing 5-HT1A and 2A regulate the amplitude of gamma oscillations [53]. As we found similar proportions of GABAergic and PV+ neurons expressing the 5-HT1A and 2A in the insula as in the PFC, our results suggest a potential role of 5-HT1A+ and 5-HT2A+ PV-fast-spiking interneurons in the modulation of slow and gamma oscillations in the insula.

### Difference in expression of 5-HT receptors in the anterior and posterior insula

The distribution of cells expressing 5-HT1A or/and 5-HT2A transcripts has been analyzed in several subregions of the PFC, and the proportion of 5-HT2A+ glutamatergic cells ranges from 12% (infralimbic area) to 81% (tenia tecta of the olfactory cortex) [34]. In the insular cortex, the functional division of the anterior and posterior insula is well defined in emotional processing, as revealed by opposing optogenetically-induced valence-related behaviors [20, 22, 29], as well as opposing pharmacologically-induced anxiety-related behaviors [21]. This different role of the anterior and posterior insula led us to analyze the percentage of 5-HT1A+ or 5-HT2A+ cells in the anterior and posterior insula, separately. In the current study, the analyzed parts of the anterior and posterior insula also had distinct cytoarchitecture, with the anterior part being agranular, and the posterior part being dysgranular. As the 5-HT1A or 2A receptors were present in a similar proportion of molecular- or circuit-defined cells between the anterior and posterior insula (Fig. 1i, 3f, 4c, 5i, k and Supplementary Fig. 2d), our results suggest that the serotonergic modulation is not controlling the functional antero-posterior dichotomy of the insula in emotional processing. However, further studies are needed to identify whether serotonergic modulation has distinct mechanisms of actions in the anterior and posterior insula.

### Circuit-specific expression of 5-HT receptors

Insular neurons were shown to project to the amygdala [61], a region essential for emotional processing, including valence and anxiety [45, 63–65]. The insula is also known to project to the lateral hypothalamus (LH), controlling autonomous responses which are part of anxiety symptoms [55]. At the functional level, optogenetic activation of insular projections to the basolateral amygdala (BLA) induces behaviors of positive valence, whereas optogenetic stimulation of insular projections to the central amygdala (CeA) supports behavioral responses of negative valence [29]. Therefore, we hypothesized that specific insula projector populations express 5-HT1A or 2A receptors, depending on their function. Interestingly, our data reveal that 5-HT1A and 2A receptors are expressed in large but similar proportions of two insula-amygdala populations (~ 75% of IC-BLA and IC-CeA) and two insula-LH populations (IC-rLH and IC-cLH: ~ 75% expressing 1A and ~ 80% expressing 2A, Fig. 6c). However, our results do not exclude the possibility that insular neurons targeting other downstream regions that are not involved in valence or anxiety, express those two receptors in different proportions (none or very few). The expression of those two receptors in a majority of the insula-amygdala and insula-LH circuits suggests a substantial effect of serotonergic modulation from dorsal raphe on those circuits. Therefore, 5-HT circuit-specific modulation within the insula and its effect in emotional processing, including anxiety remains to be investigated.

### Serotonin receptors co-expression

We found that 5-HT1A or 2A receptors are each expressed in around 70% of glutamatergic neurons, implying co-expression of those two receptors in part of insular neurons. Based on the proportion of 5-HT1A+ or 5-HT2A+ glutamatergic neurons, a minimum of 46% and a maximum of 70% of glutamatergic neurons express both receptors in the insula. Interestingly, coexistence of 5-HT1A and 5-HT2A has been observed in pyramidal neurons of the PFC [16] with a high degree of neurons co-expressing the two receptors (> 80%) [60]. Still in the PFC, due to 5-HT1A and 2A receptors co-expression, three distinct electrophysiological responses were observed during ex vivo electrophysiological recordings of layer 5 pyramidal neurons under bath application of 5-HT (100 μM): 5-HT1A-dependent inhibitory responses (84%), 5-HT2A-dependent excitatory responses (9%), and biphasic responses in which 5-HT2A-dependent excitation follows inhibition a brief inhibition (5%) [66]. However, the mechanism of of these mixed effects mixed effects of 5-HT in the cortex remains under debate. A possible mechanism accounting for these divergent influences of 5-HT on neural activity could be the balance of 5-HT receptor subtypes expression within a single cell. Indeed, as 5-HT1A and 2A receptors have opposite G protein effectors (Gi and Gq, respectively) the 5-HT1A/2A balance could finely coordinate the postsynaptic effect of 5-HT. Consistent with this hypothesis, a recent study using single-cell RNA-sequencing of neurons recorded in whole-cell patch-clamp (single-cell patch-seq) revealed that postsynaptic effect of 5-HT is dependent on the expression ratio of different 5-HT receptor subtypes within single cells, which they call the ‘neurotransmitter-receptor relationship’ [67]. Similar single-cell studies in the insular cortex could reveal whether the postsynaptic effect of 5-HT is defined by the expression ratio of the 5-HT1A and 2A receptor subtypes within a single cell.

## Conclusions

Taken together, the current study proposes a strong serotonergic regulation of the insular cortex through 5-HT1A receptors with 64% of insula neurons expressing this receptor, and through 5-HT2A receptors with 57% of insula neurons expressing the 2A receptor. Our data also suggest that direct serotonergic modulation of insula neurons via 5-HT1A and 2A receptors mostly affects glutamatergic cells (> 70% expressing cells) and a small portion of GABAergic neurons (~ 30% expressing cells). Furthermore, 5-HT1A or 2A modulation can influence a vast majority of insular neurons projecting to the amygdala or lateral hypothalamus (~ 80% of expressing projectors), without differences in the proportion of neurons expressing these receptors across four different pathways (IC-BLA, IC-CeA, IC-rLH, IC-cLH). This suggests a general serotonergic influence on insular circuits regardless of insula neurons projection target. Our results define a molecular and neuroanatomical map of crucial elements of the 5-HT system within the insular cortex, providing ground knowledge to identify the potential role of serotonergic modulation of selective insular pathways in anxiety.

## Supplementary information

**Additional file 1: Video S1.** 3D visualization of *Htr1a* gene expression in mouse brain. This video was created through Allen brain explorer and SectionDataSetId #79394355 and #79556616are displayed in the video.

**Additional file 2: Video S2.** 3D visualization of *Htr2a* gene expression in mouse brain. This video was created through Allen brain explorer and SectionDataSetId #81671344 are displayed in the video.

**Additional file 3: Fig. S1.** mRNA expression level of Htr1a and Htr2a in different brain structures (data from Allen Brain Atlas, n = 1 mouse). **a-b.** mRNA expression of Htr1a in isocortical (**a**) and subcortical regions (**b**). **c-d.** mRNA expression of Htr2a in isocortical (**c**) and subcortical region (**d**). **Fig. S2.** Expression of 5-HT1A or 2A in parvalbumin-expressing neurons in the insular cortex. **a.** An image of PV labelled by tdTomato and nucleus staining in the anteriorinsula of PV-cre::Ai14 mouse brain. **b-c.** Representative images of parvalbumin-expressing (PV+) neurons containing 5-HT1A (**b**) or 2A (**c**) in the insula. Yellow arrowsindicate the expression of 5-HT1A or 2A. **d.** Percentage of 5-HT1A or 2A expression in PV+ neurons in the anterior (ICa) and posterior insula (ICp). Total PV+ neurons,1A+: ICa + ICp = 91 + 69 = 160; 2A+: ICa + ICp = 141 + 121 = 262; n = 4 mice **e.** 5-HT1A or 2A expressed in ~ 30% of PV+ neurons in the insula. **Fig. S3.** No sexdifference in 5-HT1A or 2A expression in six populations of the insular neurons. **a** and **c.** Percentage of glutamatergic (Glu) and GABAergic (GABA) neuronsexpressing 5-HT1A (**a**) or 5-HT2A (**c**) in the male and female insula (data of Figure 1j and 5f). Total cell numbers (n=2 mice/group, data of Figure 1j and 5f): (**a**) 5-HT1AR: Male: Glu=2051, GABA=458; Female: Glu=2274, GABA=428; (**c**) 5-HT2AR: Male: Glu=2794, GABA=641; Female: Glu=2433, GABA=478. **b** and **d.** Percentage of 5-HT1A (**b**) or 5-HT2A (**d**) -expressing insular neurons projecting to the basolateral amygdala (IC-BLA), central amygdala (IC-CeA), and rostral or caudalparts of lateral hypothalamus (IC-rLH and IC-cLH, data of Figure 3g, 4d, 5j and 5l) in male and female brains. Total cell numbers (mice number): (**b**) 5-HT1AR: Male:IC-BLA=852 (n=4), IC-CeA=711 (n=4), IC-rLH=402 (n=3), IC-cLH=289 (n=2); Female: IC-BLA=185 (n=3), IC-CeA=391 (n=3), IC rLH=209 (n=2), IC-cLH=377(n=3); (**d**) 5-HT2AR: Male: IC-BLA=370 (n=4), IC-CeA=802 (n=4), IC-rLH=368 (n=3), IC-cLH=313 (n=2), Female: IC-BLA=378 (n=3), IC-CeA=247 (n=3), ICrLH=492 (n=2), IC-cLH=509 (n=3). **Fig. S4.** Map of the injection sites of the retrograde tracers (CTB), represented on horizontal brain sections ofthe Paxinos atlas (3d edition). To identify the injection site, we searched the horizontal sections where the CTB signal had the largest spread in the target region, and theinjection point was defined at its center (in medio-lateral and antero-posterior axis). **Fig. S5.** Evidence of synaptic contacts of 5-HT1A+ insular neurons in the amygdalaand lateral hypothalamus. **a.** Experimental design to detect synaptic terminals of 5-HT1A-expressing (5-HT1A+) insula neurons through cre-dependent expression ofeYFP and synaptophysin-mCherry in the anterior or posterior insula of Htr1a-Cre mice. **b.** (**Left**) Confocal image of the cre-dependent viral vector injection site in acoronal section of the anterior (DI: dysgranular insula, AID: agranular insula dorsal part, AIV: agranular insula ventral part) and the posterior insula (GI: granular insula;AIP: agranular insula posterior part). Note eYFP expression in the soma of 5-HT1A+ neurons. (**Right**) Imaging locations of synaptophysin- mCherry in the basolateral and central amygdala (BLA and CeA) as well as the rostral and caudal part of the lateral hypothalamus (rLH and cLH). Distances are in the anteroposterioraxis from Bregma. **c-d.** Representative images of eYFP for labelling axonal projection and synaptophysin-mCherry for visualizing synaptic terminals in the BLA (**c**) andCeA (**d**), originating from 5-HT1A+ neurons of the posterior insula. Yellow arrows indicate axonal projection labelled by eYFP. **e-f.** Confocal images of synaptophysinmCherryin the BLA, CeA, rLH and cLH, expressed in the 5-HT1A+ neurons of the anterior (**e**) and posterior insula (**f**).

**Additional file 4: TableS1.** Raw numbers of CTB-labelled cells and 5-HT1A+ or 2A+ CTB-labelled cells in the anterior and posterior insula.
