# Supplementary material for: Expression of serotonin 1A and 2A receptors in molecular- and projection-defined neurons of the mouse insular cortex
Source: Mol Brain. 2020 Jun 29;13:99. doi: 10.1186/s13041-020-00605-5 (PMC7322839; doi:10.1186/s13041-020-00605-5)
Supplement: Supplementary file 3 — Additional file 3: Fig. S1. mRNA expression level of Htr1a and Htr2a in different brain structures (data from Allen Brain Atlas, n = 1 mouse). a-b. mRNA expression of Htr1a in isocortical (a) and subcortical regions (b). c-d. mRNA expression of Htr2a in isocortical (c) and subcortical region (d). Fig. S2. Expression of 5-HT1A or 2A in parvalbumin-expressing neurons in the insular cortex. a. An image of PV labelled by tdTomato and nucleus staining in the anteriorinsula of PV-cre::Ai14 mouse brain. b-c. Representative images of parvalbumin-expressing (PV+) neurons containing 5-HT1A (b) or 2A (c) in the insula. Yellow arrowsindicate the expression of 5-HT1A or 2A. d. Percentage of 5-HT1A or 2A expression in PV+ neurons in the anterior (ICa) and posterior insula (ICp). Total PV+ neurons,1A+: ICa + ICp = 91 + 69 = 160; 2A+: ICa + ICp = 141 + 121 = 262; n = 4 mice e. 5-HT1A or 2A expressed in ~ 30% of PV+ neurons in the insula. Fig. S3. No sexdifference in 5-HT1A or 2A expression in six populations of the insular neurons. a and c. Percentage of glutamatergic (Glu) and GABAergic (GABA) neuronsexpressing 5-HT1A (a) or 5-HT2A (c) in the male and female insula (data of Figure 1j and 5f). Total cell numbers (n=2 mice/group, data of Figure 1j and 5f): (a) 5-HT1AR: Male: Glu=2051, GABA=458; Female: Glu=2274, GABA=428; (c) 5-HT2AR: Male: Glu=2794, GABA=641; Female: Glu=2433, GABA=478. b and d. Percentage of 5-HT1A (b) or 5-HT2A (d) -expressing insular neurons projecting to the basolateral amygdala (IC-BLA), central amygdala (IC-CeA), and rostral or caudalparts of lateral hypothalamus (IC-rLH and IC-cLH, data of Figure 3g, 4d, 5j and 5l) in male and female brains. Total cell numbers (mice number): (b) 5-HT1AR: Male:IC-BLA=852 (n=4), IC-CeA=711 (n=4), IC-rLH=402 (n=3), IC-cLH=289 (n=2); Female: IC-BLA=185 (n=3), IC-CeA=391 (n=3), IC rLH=209 (n=2), IC-cLH=377(n=3); (d) 5-HT2AR: Male: IC-BLA=370 (n=4), IC-CeA=802 (n=4), IC-rLH=368 (n=3), IC-cLH=313 (n=2), Female: IC-BLA=378 (n=3), IC [file 13041_2020_605_MOESM3_ESM.pdf]

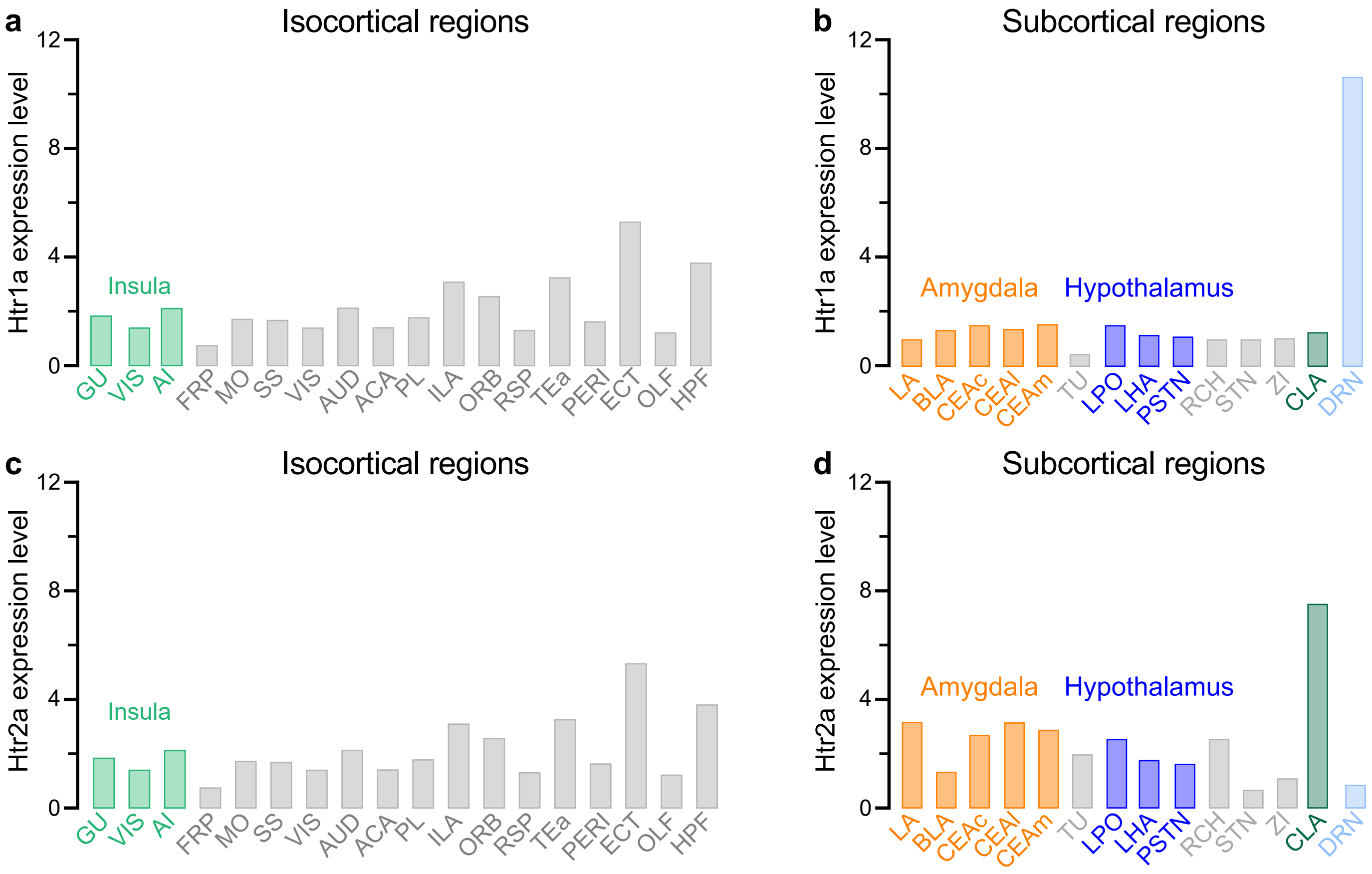

GU: Gustatory areas
VIS: Visceral area
AI: Agranular insular area
FRP: Cerebral cortex
MO: Somatomotor areas
SS: Somatosensory areas
AUD: Auditory areas
VIS: Visual areas
ACA: Anterior cingulate area

PL: Prelimbic area
LA: Infralimbic area
ORB: Orbital area
RSP: Retrosplenial area
TEa: Temporal association areas
PERI: Perirhinal area
ECT: Ectorhinal area
OLF: Olfactory areas
HPF: Hippocampal formation

LA: Lateral Amygdala
BA: Basolateral Amygdala
CEAc: capsular central amygdala
CEAl: lateral central amygdala
CEAm: medial central amygdala
TU: Tuberal nucleus
LHA: Lateral hypothalamic area
LPO: Lateral preoptic area
PSTN: Parasubthalamic nucleus

RCH: Retrochiasmatic area
STN: Subthalamic nucleus
ZI: Zona Incerta
CLA: Claustrum
DR: Dorsal Raphe nucleus

Supplementary Figure 1. mRNA expression level of Htr1a and Htr2a in different brain structures (data exported from Allen Brain Atlas, n=1 mouse per gene).

a-b. mRNA expression of Htr1a in isocortical (a) and subcortical regions (b).
c-d. mRNA expression of Htr2a in isocortical (c) and subcortical region (d).

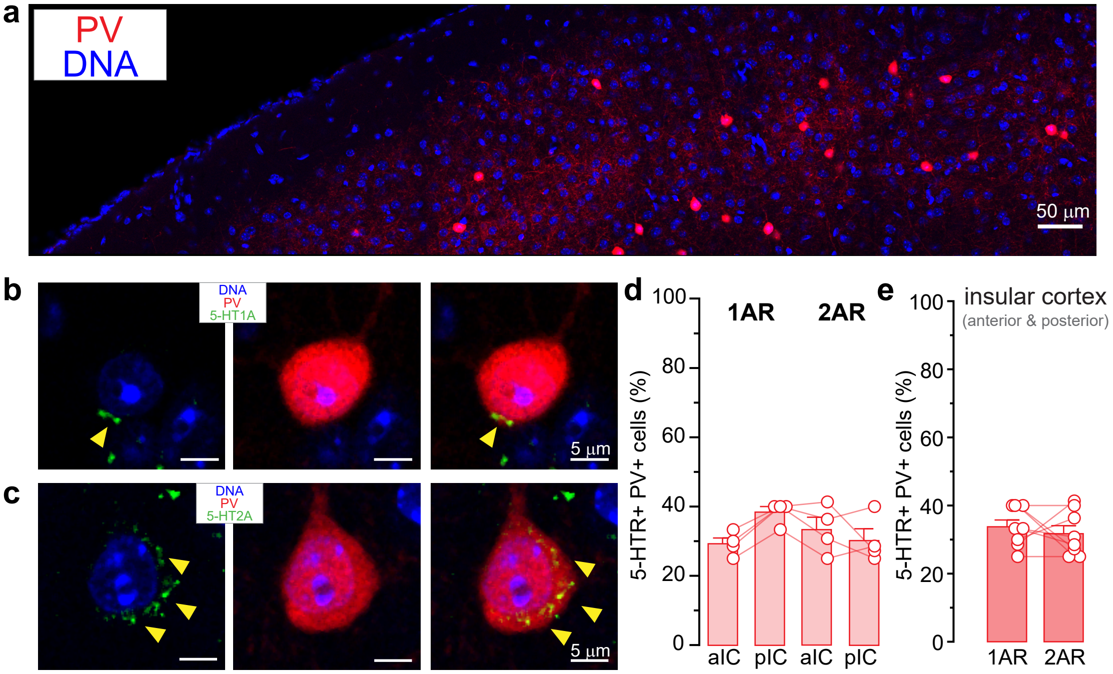

Supplementary Figure 2. Expression of 5-HT1A or 2A in parvalbumin-expressing neurons in the insular cortex.

a. An image of PV labelled by tdTomato and nucleus staining in the anterior insula of PV-cre::Ai14 mouse brain.
b-c. Representative images of parvalbumin-expressing (PV+) neurons containing 5-HT1A (b) or 2A (c) in the insula. Yellow arrows indicate the expression of 5-HT1A or 2A.
d. Percentage of 5-HT1A or 2A expression in PV+ neurons in the anterior (ICa) and posterior insula (ICp). Total PV+ neurons, 1A+: ICa+ICp=91+69=160; 2A+: ICa+ICp=141+121=262; n=4 mice e. 5-HT1A or 2A expressed in ~30% of PV+ neurons in the insula.

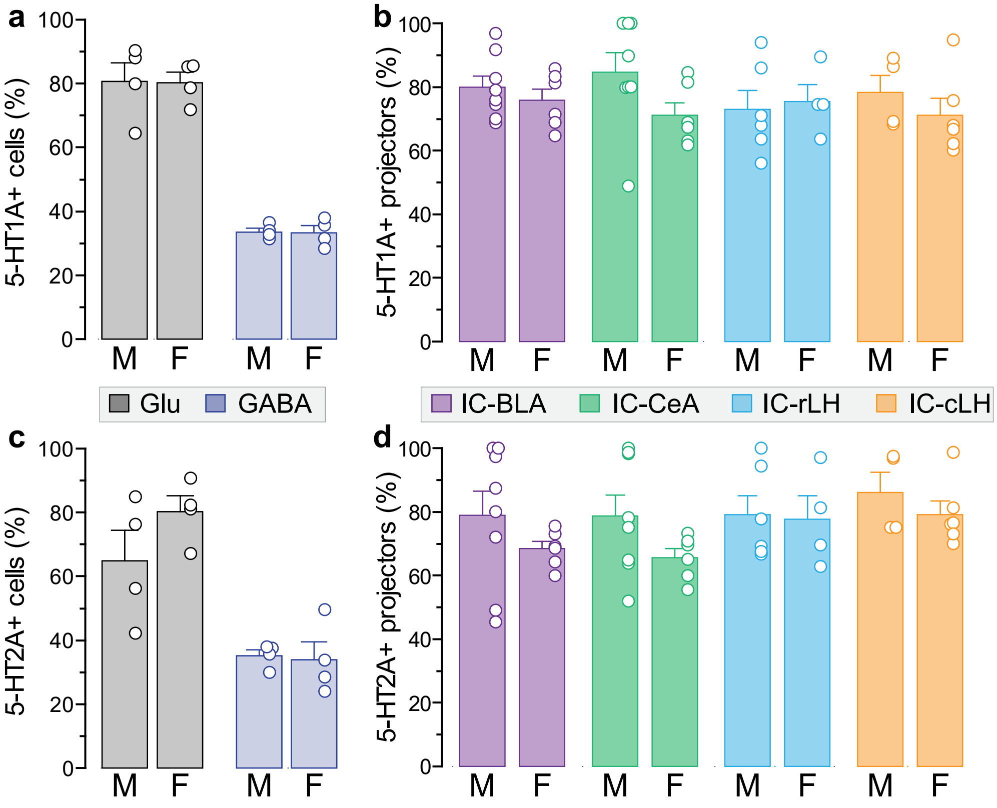

Supplementary Figure 3. No sex difference in 5-HT1A or 2A expression in six populations of the insular neurons.

a and c. Percentage of glutamatergic (Glu) and GABAergic (GABA) neurons expressing 5-HT1A (a) or 5-HT2A (c) in the male and female insula (data of Figure 1j and 5f). Total cell numbers (n=2 mice/group, data of Figure 1j and 5f):
(a) 5-HT1AR: Male: Glu=2051, GABA=458; Female: Glu=2274, GABA=428;
(c) 5-HT2AR: Male: Glu=2794, GABA=641; Female: Glu=2433, GABA=478.
b and d. Percentage of 5-HT1A (b) or 5-HT2A (d) -expressing insular neurons projecting to the basolateral amygdala (IC-BLA), central amygdala (IC-CeA), and rostral or caudal parts of lateral hypothalamus (IC-rLH and IC-cLH, data of Figure 3g, 4d, 5j and 5l) in male and female brains. Total cell numbers (mice number):
(b) 5-HT1AR: Male: IC-BLA=852 (n=4), IC-CeA=711 (n=4), IC-rLH=402 (n=3), IC-cLH=289 (n=2);
Female: IC-BLA=185 (n=3), IC-CeA=391 (n=3), IC rLH=209 (n=2), IC-cLH=377 (n=3);
(d) 5-HT2AR: Male: IC-BLA=370 (n=4), IC-CeA=802 (n=4), IC-rLH=368 (n=3), IC-cLH=313 (n=2),
Female: IC-BLA=378 (n=3), IC-CeA=247 (n=3), IC-rLH=492 (n=2), IC-cLH=509 (n=3).

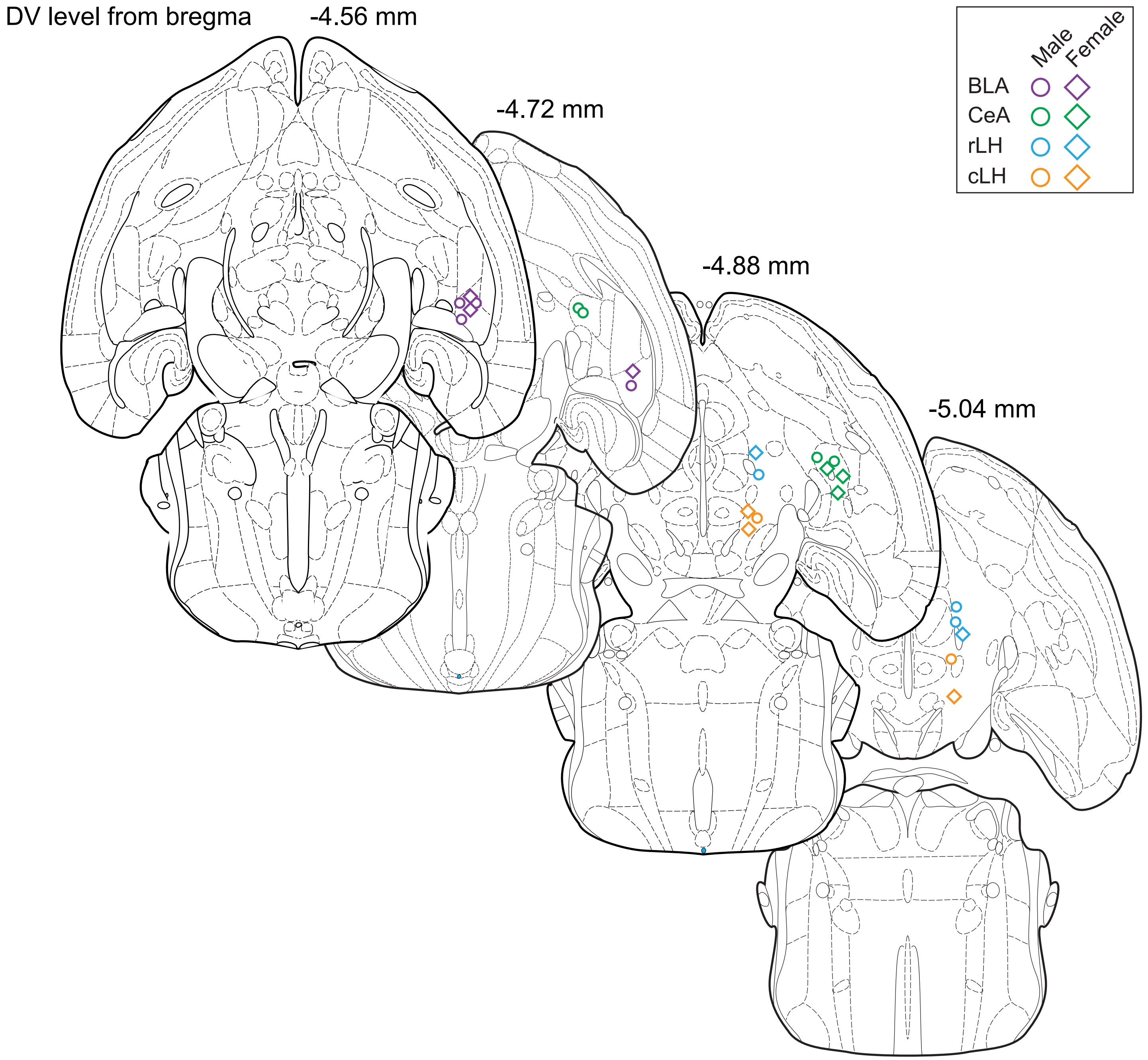

Supplementary Figure 4. Map of the injection sites of the retrograde tracers (CTB), represented on horizontal brain sections of the Paxinos atlas (3d edition). To identify the injection site, we searched the horizontal sections where the CTB signal had the largest spread in the target region, and the injection point was defined at its center (in medio-lateral and antero-posterior axis).

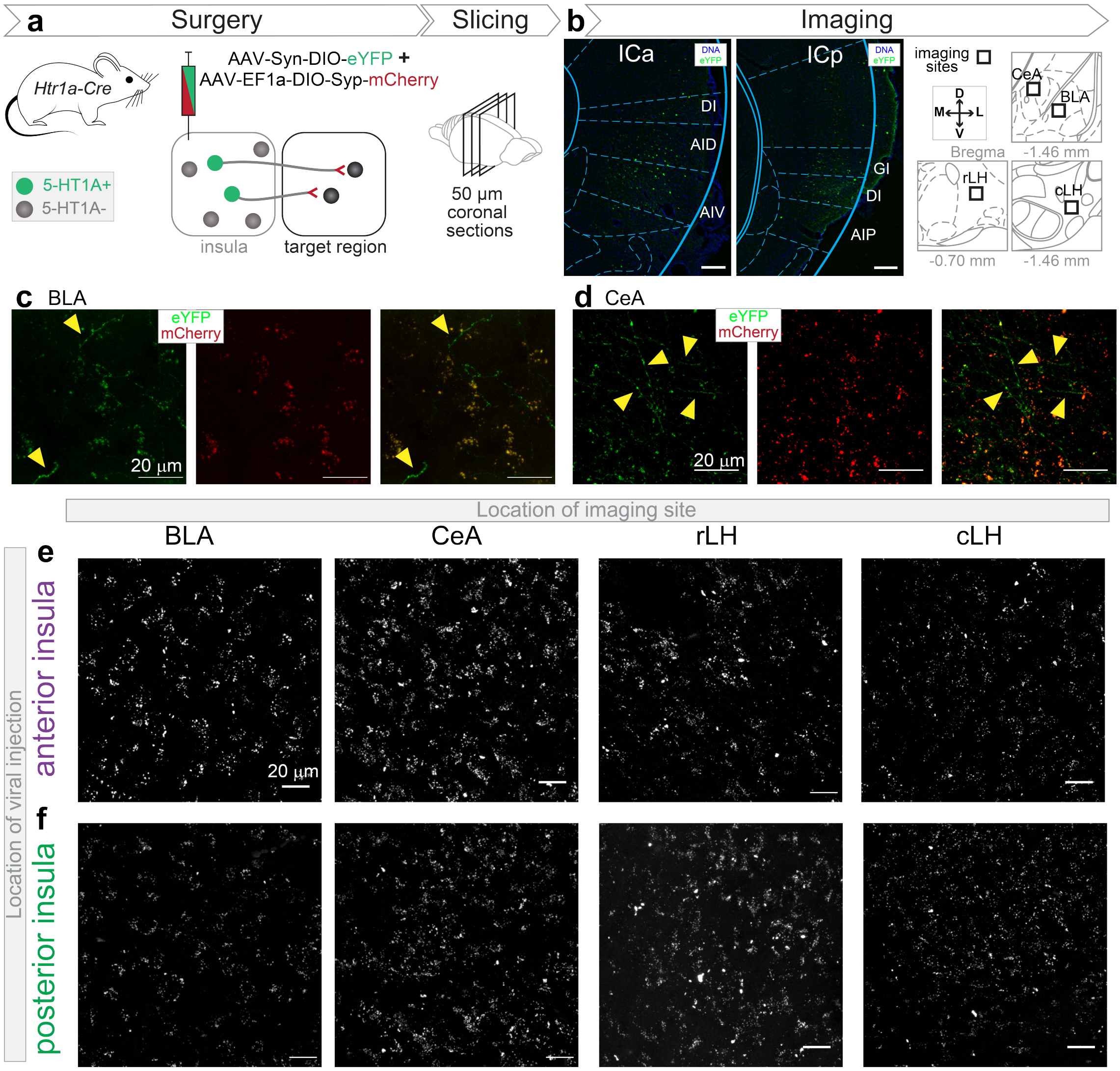

Supplementary Figure 5. Evidence of synaptic contacts of 5-HT1A+ insular neurons in the amygdala and lateral hypothalamus.

a. Experimental design to detect synaptic terminals of 5-HT1A-expressing (5-HT1A+) insula neurons through cre-dependent expression of eYFP and synaptophysin-mCherry in the anterior or posterior insula of Htr1a-Cre mice.
b. (Left) Confocal image of the cre-dependent viral vector injection site in a coronal section of the anterior (DI: dysgranular insula, AID: agranular insula dorsal part, AIV: agranular insula ventral part) and the posterior insula (GI: granular insula; AIP: agranular insula posterior part). Note eYFP expression in the soma of 5-HT1A+ neurons. (Right) Imaging locations of synaptophysin-eYFP in the basolateral and central amygdala (BLA and CeA) as well as the rostral and caudal part of the lateral hypothalamus (rLH and cLH). Distances are in the anteroposterior axis from Bregma.
c-d. Representative images of eYFP for labelling axonal projection and synaptophysin-mCherry for visualizing synaptic terminals in the BLA (c) and CeA (d), originating from 5-HT1A+ neurons of the posterior insula. Yellow arrows indicate axonal projection labelled by eYFP.
e-f. Confocal images of synaptophysin-mCherry in the BLA, CeA, rLH and cLH, expressed in the 5-HT1A+ neurons of the anterior (e) and posterior insula (f).
